# Prevention of Cardiovascular Disease and Cancer Through Early Statin Treatment in Advanced Atherosclerosis: An Observational Study

**DOI:** 10.14740/cr2196

**Published:** 2026-04-15

**Authors:** Ansgar Adams, Waldemar Bojara, Michel Romanens

**Affiliations:** a BG Prevent GmbH Zentrum Koblenz, Koblenz, Germany; b Medizinische Klinik I Kardiologie Osnabruck, Klinikum Osnabruck, Germany; c Vascular Risk Foundation (Varifo), Olten, Switzerland

**Keywords:** Microvascular dysfunction, Atherosclerosis, Cardiovascular risk, Cancer, Carotid ultrasound

## Abstract

**Background:**

The extent of atherosclerosis in healthy men and women was measured using ultrasound on the carotid artery, and it was investigated whether early treatment with statins in subjects with advanced atherosclerosis improves the outcome for cardiovascular disease and cancer.

**Method:**

From 2009 to 2017, 5,186 subjects (39.1% women) aged 35–65 with no signs of cardiovascular disease underwent ultrasound examination of the carotid artery. The total plaque area (TPA) and maximum plaque thickness were measured.

**Results:**

A follow-up was available for 4,340 (83.7%) participants. The mean follow-up period was 87 months (7.3 years) for men and 79 months (6.6 years) for women. Advanced atherosclerosis (type III, IVb) was present in 506 (11.7%) subjects. Statin treatment was initiated in 186 (36.8%) of the subjects. Events (heart attack, ischemic stroke, coronary artery bypass grafting (CABG), percutaneous transluminal coronary angioplasty (PTCA)) occurred in 170 (3.9%) of the 4,340 subjects with follow-up data. Cancer occurred in 71 (1.7%) of the subjects. The event rate for cardiovascular events was 37.6% in men with advanced atherosclerosis without statin therapy vs. 1.6% (P < 0.0001) in those with low-to-moderate plaque burden; for cancer, the rates were 8.6% vs. 1.2% (P < 0.0001). In women with advanced atherosclerosis, the event rate for cardiovascular events without statin therapy was 14.8% vs. 0.2% (P < 0.0001) in those with low-to-moderate plaque burden; and for cancer, the rates were 7.4% vs. 0.9% (P = 0.002). Treatment of male subjects with advanced atherosclerosis (type III, IVb) with a statin significantly improved the prognosis. The event rate for cardiovascular events was 16.1% in men in the treated group vs. 37.6% (P < 0.0001) in the untreated group, and 2.7% vs. 14.8% (P = 0.077) in women. The event rate for cancer was 2% in men in the treated group vs. 8.6% (P = 0.006) in the untreated group. Due to the low number of cases in women, a statistical evaluation did not make sense. Mortality (from any cause) was significantly lower in men treated with statins (P = 0.008).

**Conclusions:**

Treatment with statins in subjects with advanced atherosclerosis of the carotid artery (type III, IVb findings on ultrasound) significantly improved the prognosis in a non-randomized observational study.

## Introduction

Previous studies have shown that advanced atherosclerosis of the carotid artery is associated with an increased risk of cardiovascular disease. There is evidence that there is also an increased risk of cancer [1–13]. By measuring the total plaque area (TPA) and the maximum plaque thickness in the carotid artery, individuals with a high cardiovascular risk can be identified. Early treatment with statins improves the outcome for cardiovascular disease [14]. The aim of the study was to investigate whether there is a link between advanced atherosclerosis of the carotid artery and the incidence of cancer, and whether the risk can be reduced by statin treatment.

## Materials and Methods

### Study design

This was a retrospective study. All described investigations and evaluations on humans were conducted with the approval of the responsible ethics committee. The study was conducted in compliance with the ethical standards of the responsible institution on human subjects as well as with the Helsinki Declaration.

### Study population

Between 2009 and 2017, heart attack risk assessment was offered as part of occupational health screening in companies in various industries (chemical, glass, pharmaceutical, administration, metal, social services, paper, printing, ceramics, information technology (IT), universities, technical colleges, retail) in the Koblenz region. A total of 5,186 test subjects (39.1% women) aged between 35 and 65 with no signs of cardiovascular disease underwent ultrasound examinations of both carotid arteries. Follow-up data was available for 4,340 (83.7%) test subjects. A portable ultrasound device from Kontron Medical, type Imagic Agile, with a 10 MHz linear scanner was used.

### Carotid plaque measurements

The measurement method was performed as previously published [14]. The classification of ultrasound findings was developed on an independent group of patients who were examined 1 day before a planned coronary angiography. Low risk corresponds to ultrasound findings of type I and IIa, medium risk to findings of type IIb and IVa, and high risk to findings of type III and IVb [15].

### Clinical intervention and patient management

High-risk patients (ultrasound findings type III and IVb) received a letter to their family doctor to start treatment with a statin, such as atorvastatin 10–20 mg. One hundred forty-nine (35.9%) men and 37 (40.7%) women received a statin. Twelve subjects took statins irregularly. If the duration of use was less than 50% of the follow-up period, this was considered no treatment, and *vice versa*. Seven subjects did not tolerate statins and had to discontinue use. The authors had no influence on whether the primary care physician prescribed a statin or not. After 4–6 weeks, it was determined whether statin treatment had been initiated, and at each follow-up examination, questions were asked about any cardiovascular events or cancer. In the first years after the start of the study in 2009, the acceptance rate for initiating statin therapy among general practitioners was only 36.8%. In recent years (e.g., 2020–2026), the rate of treated patients has increased to approximately 75%.

### Statistical analysis

The baseline characteristics of all subjects were described according to risk groups (low, medium, high)—continuous scale: mean, standard deviation; categorical scale: absolute and relative frequencies—and compared using the *t*-test, χ^2^ test, or Fisher’s exact test.

## Results

A total of 3,158 men and 2,028 women underwent ultrasound examination. Follow-up data were available for 2,512 (79.5%) men and 1,828 (90.1%) women. Advanced atherosclerosis was present in 415 (16.5%) men and 91 (5.0%) women. The mean follow-up period was 87 months for men and 79 months for women (Table 1). There were 158 cardiovascular events in men (strokes (n = 23), heart attacks (n = 67), coronary artery bypass grafting (CABG, n = 29), and percutaneous transluminal coronary angioplasty (PTCA, n = 39). Advanced atherosclerosis was present in 124 (78.5%) of the men and was thus correctly predicted. Cancer occurred in 52 (2.1%) of the men (prostate (n = 11), bronchial (n = 5), colon (n = 5), stomach (n = 4), unknown (n = 4), leukemia (n = 3), bladder (n = 3), kidney (n = 2), pancreas (n = 2), myeloma (n = 2), brain (n = 2), testicular (n = 2), melanoma (n = 1), Hodgkin’s disease (n = 1), esophageal (n = 1), laryngeal (n = 1), tongue base (n = 1), lymphoma (n = 1), liver (n = 1)).

**Table 1:** Baseline Characteristics of All Subjects by Risk Groups

Characteristics	Male, type I, IIa, IIb, IVa	Male, type III, IVb	P value	Female, type I, IIa, IIb, IVa	Female type III, IVb	P value
N	2,097	415		1,737	91	
Age	48 ± 6	54 ± 6	< 0.0001^a^	48 ± 7	53 ± 6	< 0.0001^a^
Body mass index (BMI), kg/m^2^	27.31 ± 4.36	27.93 ± 3.81	0.004^a^	25.19 ± 4.68	25.29 ± 4.3	0.834^a^
Current smoker	473 (22.6%)	189 (45.5%)	< 0.0001^a^	357 (20.0%)	45 (49.5%)	< 0.0001^a^
LDL cholesterol, mg/dL	149 ± 52	159 ± 32	< 0.0001^a^	141 ± 33	157 ± 38	0.0007^a^
HDL cholesterol, mg/dL	51 ± 12	48 ± 10	0.0004^a^	65 ± 15	62 ± 16	0.095^a^
Triglycerides, mg/dL	168 ± 123	196 ± 126	0.0001^a^	109 ± 58	135 ± 62	0.0019^a^
SBP, mm Hg	127 ± 15	135 ± 20	< 0.0001^a^	121 ± 16	129 ± 20	< 0.0001^a^
DBP, mm Hg	81 ± 8	84 ± 10	< 0.0001^a^	78 ± 9	79 ± 14	0.181^a^
Diabetes mellitus	65 (3.1%)	35 (8.4%)	< 0.0001^b^	25 (1.4%)	5 (5.5%)	0.0145^c^
Antihypertensive therapy	384 (18.3%)	142 (34.2%)	< 0.0001^a^	280 (16.1%)	38 (41.8%)	< 0.0001^b^
Positive family history	448 (21.4%)	116 (28.0%)	0.006^a^	478 (27.5%)	40 (44.0%)	< 0.0001^b^
Tot plaque area (TPA), mm^2^	29 ± 61	149 ± 65	< 0.0001^a^	17 ± 24	119 ± 55	< 0.0001^a^
Plaque thickness (Max), mm	1.7 ± 0.7	2.9 ± 0.7	< 0.0001^a^	1.6 ± 0.4	3.1 ± 0.6	< 0.0001^a^
Event (MACE, CABG, PTCA)	34 (1.6%)	124 (29.9%)	< 0.0001^b^	3 (0.2%)	9 (9.9%)	< 0.0001^c^
Event MACE	21 (1.0%)	69 (16.6%)	< 0.0002^b^	2 (0.1%)	5 (5.5%)	< 0.0001^c^
Carcinoma	26 (1.2%)	26 (6.3%)	< 0.0001^b^	15 (0.9%)	4 (4.4%)	0.013^c^
Death, all-cause	15 (0.7%)	42 (10.1%)	< 0.0001^b^	4 (0.2%)	1 (1.1%)	0.225^c^
Death, CVD	2 (0.1%)	22 (5.3%)	< 0.0001^c^	1 (0.07%)	0	1^c^
Death, carcinoma	5 (0.2%)	15 (3.6%)	< 0.0001^c^	1 (0.07%)	1 (1.1%)	0.0097^c^
Follow-up month (Min–Max)	87 (2–195)	80 (2–176)	0.002^a^	79 (2–186)	78 (2–163)	0.805^a^

The mean values with the standard deviations or the percentages are shown. ^a^*t*-test; ^b^χ^2^ test; ^c^Fish’s exact test. BMI: body mass index; CABG: coronary artery bypass grafting; CVD: cardiovascular disease; DBP: diastolic blood pressure; HDL: high-density lipoprotein; LDL: low-density lipoprotein; MACE: major adverse cardiovascular event; PTCA: percutaneous transluminal coronary angioplasty; SBP: systolic blood pressure; Min: minimum; Max: maximum.

Twelve cardiovascular events occurred in women (strokes (n = 3), heart attacks (n = 4), CABG (n = 1), and PTCA (n = 4)). Nine (75%) of the women had advanced atherosclerosis, which was correctly predicted. Among women, there were 19 (0.9%) cases of cancer (breast (n = 11), pancreatic (n = 2), colon (n = 1), bile duct (n = 1), uterine (n = 1), bronchial (n = 1), stomach (n = 1), unknown (n = 1)) (Table 1).

Treatment of male subjects with advanced atherosclerosis (type III, IVb) with a statin significantly improved the prognosis. The event rate for cardiovascular events in men was 16.1% in the treated group versus 37.6% (P < 0.0001) in the untreated group; and the event rate for cancer was 2% in the treated group versus 8.6% (P < 0.006) in the untreated group (Table 2). The event rate for cardiovascular events in women was 2.7% in the treated group versus 14.8% (P = 0.077) in the untreated group. Due to the low number of cancers in women, a statistical evaluation did not make sense (Table 3).

**Table 2:** Baseline Characteristics of All Men With Type III and IVb

Characteristics	Male, type III, IVb without statin therapy	Male, type III, IVb with statin therapy	P value
N	266	149	
Age	53 ± 6	54 ± 6	0.056^a^
Body mass index (BMI), kg/m^2^	27.60 ± 3.81	28.20 ± 4.31	0.144^a^
Current smoker	129 (48.5%)	60 (40.3%)	0.123^b^
LDL cholesterol, mg/dL	157 ± 32	161 ± 49	0.351^a^
HDL cholesterol, mg/dL	49 ± 10	47 ± 11	0.254^a^
Triglycerides, mg/dL	197 ± 126	193 ± 127	0.789^a^
SBP, mm Hg	134 ± 20	133 ± 15	0.478^a^
DBP, mm Hg	84 ± 10	82 ± 8	0.014^a^
Diabetes mellitus	20 (7.6%)	15 (10.0%)	0.364^b^
Antihypertensive therapy	75 (28.2%)	67 (45.0%)	0.0006^b^
Positive family history	76 (28.6%)	40 (26.8%)	0.707^b^
Tot plaque area (TPA), mm^2^	146 ± 65	152 ± 59	0.229^a^
Plaque thickness (Max), mm	2.9 ± 0.7	3.0 ± 0.8	0.151^a^
Event (MACE, CABG, PTCA)	100 (37.6%)	24 (16.1%)	< 0.0001^b^
Event MACE	59 (22.2%)	10 (6.7%)	< 0.0001^b^
Carcinoma	23 (8.6%)	3 (2.0%)	0.0059^c^
Death, all-cause	33 (12.4%)	9 (6.0%)	0.042^c^
Death, CVD	18 (6.8%)	4 (2.7%)	0.108^c^
Death, carcinoma	13 (4.9%)	2 (1.3%)	0.097^c^
Follow-up month (Min–Max)	75 (2–176)	88 (3–170)	0.001^a^

The mean values with the standard deviations or the percentages are shown. ^a^*t*-test; ^b^χ^2^ test; ^c^Fish’s exact test. BMI: body mass index; CABG: coronary artery bypass grafting; CVD: cardiovascular disease; DBP: diastolic blood pressure; HDL: high-density lipoprotein; LDL: low-density lipoprotein; MACE: major adverse cardiovascular event; PTCA: percutaneous transluminal coronary angioplasty; SBP: systolic blood pressure; Min: minimum; Max: maximum.

**Table 3:** Baseline Characteristics of All Women With Type III and IVb

Characteristics	Female type III, IVb without statin therapy	Female type III, IVb with statin therapy	P value
N	54	37	
Age	52 ± 6	55 ± 6	0.052^a^
Body mass index (BMI), kg/m^2^	25.30 ± 4.3	25.27 ± 5.3	0.978^a^
Current smoker	26 (48.1%)	19 (51.4%)	0.764^b^
LDL cholesterol, mg/dL	158 ± 38	156 ± 43	0.816^a^
HDL cholesterol, mg/dL	64 ± 16	60 ± 12	0.246^a^
Triglycerides, mg/dL	129 ± 62	143 ± 79	0.372^a^
SBP, mm Hg	132 ± 20	126 ± 18	0.151^a^
DBP, mm Hg	81 ± 14	79 ± 8	0.153^a^
Diabetes mellitus	2 (3.7%)	3 (8.1%)	0.393^c^
Antihypertensive therapy	20 (37.0%)	18 (48.6%)	0.269^b^
Positive family history	26 (48.1%)	14 (37.8%)	0.33^b^
Tot plaque area (TPA), mm^2^	112 ± 55	129 ± 72	0.208^a^
Plaque thickness (Max), mm	3.1 ± 0.6	3.1 ± 0.6	0.996^a^
Event (MACE, CABG, PTCA)	8 (14.8%)	1 (2.7%)	0.077^c^
Event MACE	4 (7.4%)	1 (2.7%)	0.644^c^
Carcinoma	4 (7.4%)	0	0.143^c^
Death, all-cause	1 (1.7%)	0	1^c^
Death CVD	0	0	1^c^
Death carcinoma	1 (1.7%)	0	1^c^
Follow-up month (Min–Max)	71 (2–163)	89 (5–161)	0.027^a^

The mean values with the standard deviations or the percentages are shown. ^a^*t*-test; ^b^χ^2^ test; ^c^Fish’s exact test. BMI: body mass index; CABG: coronary artery bypass grafting; CVD: cardiovascular disease; DBP: diastolic blood pressure; HDL: high-density lipoprotein; LDL: low-density lipoprotein; MACE: major adverse cardiovascular event; PTCA: percutaneous transluminal coronary angioplasty; SBP: systolic blood pressure; Min: minimum; Max: maximum.

The event rate for cardiovascular events was 37.6% in men with advanced atherosclerosis without statin therapy vs. 1.6% (P < 0.0001) in those with low-to-moderate plaque burden, and for cancer, the rates were 8.6% vs. 1.3% (P < 0.0001) (Table 4). In women with advanced atherosclerosis, the event rate for cardiovascular events without statin therapy was 14.8% vs. 0.2% (P < 0.0001) in those with low-to-moderate plaque burden, and for cancer, the rates were 7.4% vs. 0.9% (P = 0.002) (Table 5). Mortality (from all causes) was significantly lower in men treated with statins (P = 0.008) (Table 6). Due to the low number of cancers in women, a statistical evaluation did not make sense (Table 7).

**Table 4:** Baseline Characteristics of All Men With Type I–IVa and Type III and IVb Without Therapy

Characteristics	Male, type I, IIa, IIb, IVa	Male, type III, IVb without statin therapy	P value
N	2,097	266	
Age	48 ± 6	53 ± 6	< 0.0001^a^
Body mass index (BMI), kg/m^2^	27.31 ± 4.36	27.60 ± 3.81	0.134^a^
Current smoker	473 (22.6%)	129 (48.5%)	< 0.00001^b^
LDL cholesterol, mg/dL	149 ± 52	157 ± 32	0.0001^a^
HDL cholesterol, mg/dL	51 ± 12	49 ± 10	0.018^a^
Triglycerides, mg/dL	168 ± 123	197 ± 126	0.0008^a^
SBP, mm Hg	127 ± 15	134 ± 20	< 0.00001^a^
DBP, mm Hg	81 ± 8	84 ± 10	< 0.00001^a^
Diabetes mellitus	65 (3.1%)	20 (7.6%)	0.0002^b^
Antihypertensive therapy	384 (18.3%)	75 (28.2%)	0.0001^b^
Positive family history	448 (21.4%)	76 (28.6%)	0.0077^b^
Tot plaque area (TPA), mm^2^	29 ± 61	146 ± 65	< 0.00001^a^
Plaque thickness (Max), mm	1.7 ± 0.7	2.9 ± 0.7	< 0.00001^a^
Event (MACE, CABG, PTCA)	34 (1.6%)	100 (37.6%)	< 0.0001^b^
Event MACE	21 (1.0%)	59 (22.2%)	< 0.0001^b^
Carcinoma	26 (1.2%)	23 (8.6%)	< 0.0001^b^
Death, all-cause	15 (0.7%)	33 (12.4%)	< 0.0001^b^
Death, CVD	2 (0.1%)	18 (6.8%)	< 0.0001^c^
Death, carcinoma	5 (0.2%)	13 (4.9%)	< 0.0001^c^
Follow-up month (Min–Max)	87 (2–195)	75 (2–176)	< 0.00001^a^

The mean values with the standard deviations or the percentages are shown. ^a^*t*-test; ^b^χ^2^ test; ^c^Fish’s exact test. BMI: body mass index; CABG: coronary artery bypass grafting; CVD: cardiovascular disease; DBP: diastolic blood pressure; HDL: high-density lipoprotein; LDL: low-density lipoprotein; MACE: major adverse cardiovascular event; PTCA: percutaneous transluminal coronary angioplasty; SBP: systolic blood pressure; Min: minimum; Max: maximum.

**Table 5:** Baseline Characteristics of All Women With Type I–IVa and Type III and IVb Without Therapy

Characteristics	Female, type I, IIa, IIb, IVa	Female type III, IVb without statin therapy	P value
N	1,737	54	
Age	48 ± 7	52 ± 6	0.0443^a^
Body mass index (BMI), kg/m^2^	25.19 ± 4.68	25.30 ± 4.3	0.843^a^
Current smoker	357 (20.0%)	26 (48.1%)	< 0.00001^b^
LDL cholesterol, mg/dL	141 ± 33	158 ± 38	0.0043^a^
HDL cholesterol, mg/dL	65 ± 15	64 ± 16	0.583^a^
Triglycerides, mg/dL	109 ± 58	129 ± 62	0.045^a^
SBP, mm Hg	121 ± 16	132 ± 20	0.0001^a^
DBP, mm Hg	78 ± 9	81 ± 14	0.0015^a^
Diabetes mellitus	25 (1.4%)	2 (3.7%)	0.194^c^
Antihypertensive therapy	280 (16.1%)	20 (37.0%)	< 0.0001^b^
Positive family history	478 (27.5%)	26 (48.1%)	0.0009^b^
Tot plaque area (TPA), mm^2^	17 ± 24	112 ± 55	< 0.0001^a^
Plaque thickness (Max), mm	1.6 ± 0.4	3.1 ± 0.6	< 0.0001^a^
Event (MACE, CABG, PTCA)	3 (0.2%)	8 (14.8%)	< 0.0001^c^
Event MACE	2 (0.1%)	4 (7.4%)	< 0.0001^c^
Carcinoma	15 (0.9%)	4 (7.4%)	0.002^c^
Death, all-cause	4 (0.2%)	1 (1.9%)	0.1421^c^
Death, CVD	1 (0.07%)	0	1^c^
Death, carcinoma	1 (0.07%)	1 (1.9%)	0.059^c^
Follow-up month (Min–Max)	79 (2–186)	71 (2–163)	0.152^a^

The mean values with the standard deviations or the percentages are shown. ^a^*t*-test; ^b^χ^2^ test; ^c^Fish’s exact test. BMI: body mass index; CABG: coronary artery bypass grafting; CVD: cardiovascular disease; DBP: diastolic blood pressure; HDL: high-density lipoprotein; LDL: low-density lipoprotein; MACE: major adverse cardiovascular event; PTCA: percutaneous transluminal coronary angioplasty; SBP: systolic blood pressure; Min: minimum; Max: maximum.

**Table 6:** Mortality of All Men With and Without Statin Treatment (Including Low and Medium Risk)

Characteristics	Male type I, IIa, IIb, IVa, IVb with statin therapy	Male type I, IIa, IIb, IVa, IVb without statin therapy	P value
N	2,246	2,363	
Death, all-cause	24 (01.1%)	48 (2.0%)	0.0084^a^
Death, CVD	6 (0.3%)	20 (0.8%)	0.0097^b^
Death, carcinoma	7(0.3%)	18 (0.8%)	0.0444^b^

^a^χ^2^ test; ^b^Fish’s exact test. CVD: cardiovascular disease.

**Table 7:** Mortality of All Women With and Without Statin Treatment (Including Low and Medium Risk)

Characteristics	Women type I, IIa, IIb, IVa, IVb with statin therapy	Women type I, IIa, IIb, IVa, IVb without statin therapy	P value
N	1,774	1,791	
Death, all-cause	4 (0.2%)	5 (0.3%)	1^a^
Death CVD	1 (0.3%)	1 (0.06%)	1^a^
Death carcinoma	1 (0.3%)	1 (0.06%)	1^a^

^a^Fish’s exact test. CVD: cardiovascular disease.

Due to the low event rates for cardiovascular disease and cancer, the low- and medium-risk groups (types I, IIa, and IIb, IVa) were combined.

## Discussion

Atherosclerosis is a disease that develops in vessels supplied by vasa vasorum, such as coronary vessels, aorta, carotid artery, and femoral arteries. Smaller arteries supplied by diffusion from the vessel lumen, such as the mammary artery or radial artery, are not or very rare affected [16]. Plaques probably develop as a result of microvascular dysfunction of the vasa vasorum and arterioles, leading to hypoxia in various organs (heart muscle with coronary microvascular dysfunction (CMD), kidney with chronic kidney disease (CKD), cerebral vessels with cerebral small vessel disease (CSVD), peripheral arterial disease (PAD), skin with systemic sclerosis, retinopathy, etc.) [17, 18]. The development of microvascular dysfunction leads to functional changes (increased vascular tone due to increased production of endothelin, prostacyclin, peptide Y, or decreased production of nitric oxide NO) and structural changes with obstruction and perivascular fibrosis. Furthermore, there is evidence that microvascular dysfunction also increases the risk of cancer [19, 20]. Microvascular dysfunction causes hypoxia, which promotes the development of atherosclerosis with the clinical manifestation of cardiovascular disease and the development of cancer. Hypoxia can downregulate the immune system and thereby slow down tumor defense. It also affects the proliferation and gene expression in tumor cells, including hypoxia-inducible factor-1 alpha (HIF-1α). Natural killer (NK) cells are the first line of defense against infections and cancer cells. Hypoxia upregulates HIF-1α, which slows down the activity of NK cells in tumor defense [21, 22]. Treatment with statins leads to an improvement in microvascular function [23–27] and a reduction in cardiovascular events. Several studies described a preventive effect of statin therapy for cancer [28–33].

The study showed that subjects with advanced atherosclerosis (type III, IVb on ultrasound) had a significantly higher risk of cardiovascular disease (MACE, CABG, PTCA) (men: 29.9% vs. 1.6%, P < 0.0001; women: 9.9% vs. 0.2%, P < 0.0001) and cancer (6.3% vs. 1.2%, P ≤ 0.0001 in men and 4.4% vs. 0.9%, P = 0.013 in women) compared to those with low-to-moderate exposure.

Early treatment of male subjects with advanced atherosclerosis with statins improved the prognosis for MACE, CABG, PTCA (event rate 16.1% vs. 37.6%, P < 0.0001) and for carcinomas (event rate 2% vs. 8.6%, P < 0.0059). Due to the low number of cases, further research is certainly needed here. In female subjects, statistical analysis was not meaningful due to the low number of cases.

The results of the study support the hypothesis that there is a link between microvascular dysfunction and the development of atherosclerosis and cancer. This could also explain the preventive effect of statin treatment, which contributes to an improvement in microvascular function.

### Limitations

This is an observational study rather than a randomized study. The disadvantage of non-randomized studies is that biases can occur, which cannot be ruled out. Furthermore, there is no analysis of whether atorvastatin was always prescribed and, if so, at what dose. The treated and untreated groups of men with advanced atherosclerosis did not differ statistically in baseline values, except for diastolic blood pressure. Nevertheless, it is possible that the occurrence of cancer influenced the use of a statin. Another limitation is that the treatment rate was very low, at approximately 37%. The follow-up rate is reduced to 87.3% because the examination was only performed once in some companies and therefore no follow-up is available.

Whether treatment of advanced atherosclerosis with statins leads to a reduction in cancer incidence needs to be verified by further studies. It would be useful to investigate this retrospectively in large plaque and coronary artery calcium (CAC) studies that have already been conducted.

### Conclusions

Carotid screening with ultrasound is useful because it allows subjects with advanced disease to be identified at an early stage and then treated. Treatment with statins in subjects with advanced atherosclerosis of the carotid artery (type III, IVb findings on ultrasound) significantly improved the prognosis for cardiovascular diseases in a non-randomized observational study. The observation that patients with advanced atherosclerosis develop cancer significantly more often than people with mild or moderate atherosclerosis is new, and that this risk can be significantly reduced under statin therapy, at least in men.

## Data Availability

Any inquiries regarding supporting data availability of this study should be directed to the corresponding author.
